# Prognostic Value of C-Reactive Protein in Adults With Tuberculous Meningitis: A Prospective Cohort Study

**DOI:** 10.1093/cid/ciaf261

**Published:** 2025-06-24

**Authors:** Lillian Tugume, Fiona V Cresswell, Nicole W Engen, Asmus Tukundane, Sarah Kimuda, Timothy Mugabi, Suzan Namombwe, Enock Kagimu, Mable Kabahubya, Jayne Ellis, Nathan C Bahr, David B Meya, David R Boulware

**Affiliations:** College of Health Sciences, Infectious Diseases Institute, Makerere University, Kampala Uganda; College of Health Sciences, Infectious Diseases Institute, Makerere University, Kampala Uganda; Department of HIV Interventions, MRC/UVRI-LSHTM Uganda Research Unit, Entebbe, Uganda; Center for Global Health and Infection, Brighton and Sussex Medical School, Brighton, United Kingdom; Division of Biostatistics and Health Data Science, University of Minnesota, Minneapolis, Minnesota, USA; College of Health Sciences, Infectious Diseases Institute, Makerere University, Kampala Uganda; College of Health Sciences, Infectious Diseases Institute, Makerere University, Kampala Uganda; College of Health Sciences, Infectious Diseases Institute, Makerere University, Kampala Uganda; College of Health Sciences, Infectious Diseases Institute, Makerere University, Kampala Uganda; College of Health Sciences, Infectious Diseases Institute, Makerere University, Kampala Uganda; College of Health Sciences, Infectious Diseases Institute, Makerere University, Kampala Uganda; College of Health Sciences, Infectious Diseases Institute, Makerere University, Kampala Uganda; Clinical Research Department, London School of Hygiene and Tropical Medicine, London, United Kingdom; Division of Infectious Diseases & International Medicine, Department of Medicine, University of Minnesota, Minneapolis, Minnesota, USA; College of Health Sciences, Infectious Diseases Institute, Makerere University, Kampala Uganda; Division of Infectious Diseases & International Medicine, Department of Medicine, University of Minnesota, Minneapolis, Minnesota, USA; Department of Medicine, Makerere University College of Health Sciences, Kampala, Uganda; Division of Infectious Diseases & International Medicine, Department of Medicine, University of Minnesota, Minneapolis, Minnesota, USA

**Keywords:** meningitis, tuberculosis, C-reactive protein, HIV, tuberculous meningitis

## Abstract

We enrolled 135 adults with tuberculous meningitis (TBM), including 83% with HIV. Participants with baseline C-reactive protein (CRP) ≥40 mg/L had 3 times higher odds of an 8-week modified Rankin scale ≥4 (adjusted odds ratio, 2.78; 95% CI: 1.28–6.04; *P* = .010). CRP is a viable prognostic biomarker in TBM.

## Teaser

Among a cohort of adults with tuberculous meningitis, including 83% living with human immunodeficiency virus, we found that baseline C-reactive protein ≥40 mg/L independently predicts 3 times higher odds of severe disability or death at 8 weeks of antituberculous therapy.

Tuberculous meningitis (TBM) comprises approximately 5% of tuberculosis, with a case fatality of approximately 50% in people with human immunodeficiency virus (HIV) [1, 2]. Mortality is 2-fold higher in adults with HIV versus adults without HIV, and survival is frequently accompanied by neurological disability [2, 3].

TBM pathology is largely attributed to a dysregulated host inflammatory response as TBM is classically a paucibacillary disease [4]. Induction of meningitis and death by nonviable bacilli has been previously demonstrated in animal studies [5]. Accordingly, host-directed immunomodulatory therapy is a priority research area for improving clinical outcomes. The current evidence shows that adjunctive dexamethasone confers an approximately 30% reduction in mortality among individuals without HIV [6]. Whereas, for adults with HIV-associated TBM, adjunctive dexamethasone resulted in a nonstatistically significant 5% absolute reduction in 12-month mortality [7]. The differential lack of benefit of adjunctive dexamethasone in HIV-associated TBM may be explained by heterogeneity of the inflammatory response.

Within the HIV-associated TBM population, subgroups with heterogeneous inflammatory responses likely exist. Some display a hyperinflammatory phenotype, and others are hypoinflammatory per the damage-response framework [8, 9]. This is exemplified by approximately 25% of individuals with HIV-associated TBM presenting with marked cerebrospinal fluid (CSF) lymphocytic pleocytosis (>250 white cells/μL), while another approximately 25% display acellular CSF (<5 white cells/μL) [10]. The subgroup with heightened dysregulated inflammation could benefit the most from host-directed therapy, while those with anergy may derive harm from antiinflammatory agents.

A low-cost biomarker that is sensitive to inflammation and predicts survival would be a promising tool for investigating customized immunomodulatory therapy for HIV-associated TBM. We sought to determine whether baseline CRP levels in blood predict poor clinical outcomes in TBM.

## METHODS

### Setting and Participants

We enrolled a cohort of Ugandan adults (aged ≥18 years) with TBM from March 2021 to November 2023 at Mulago and Kiruddu national referral hospitals in Kampala, Uganda. We included patients with TBM per the uniform case definition [11]. TBM microbiological confirmation used positive CSF Xpert MTB/RIF Ultra (Cepheid, Sunnyvale, CA) or CSF mycobacterial growth indicator tube culture (Becton Dickinson, Franklin Lakes, NJ). Individuals who presented within 3 months of starting antiretroviral therapy were classified as unmasking immune reconstitution inflammatory syndrome (IRIS).

### Procedures

Participants received antituberculous therapy via the HARVEST trial, a phase 3 multisite randomized clinical trial investigating the efficacy of high-dose oral rifampicin (35 mg/kg/d) for 8 weeks versus standard-of-care rifampicin (10 mg/kg/d) [12]. All participants received dexamethasone 0.4 mg/kg/d, weaned over 6 weeks. We conducted CRP testing on baseline serum, generally collected within 24 hours of diagnostic lumbar punctures. All HARVEST participants with an available blood sample for CRP testing were included. We measured CRP using a Cobas c311 clinical chemistry analyzer (Roche, Basel, Switzerland). Functional status at 8 weeks of antitubercular therapy was measured using the modified Rankin scale (modified Rankin) [13], which ranges from 0 (no disability) to 6 (dead) and is widely used for measuring global disability after stroke or other causes of neurologic disability.

All participants or their surrogates provided written informed consent. The Mulago Hospital Regulatory and Ethics Committee and Uganda National Council of Science and Technology approved the observational cohort and HARVEST trial.

### Statistical Analyses

We included participants who had baseline CRP and outcome status at 8 weeks in the analysis. We performed a receiver operating characteristic curve (ROC) analysis to assess serum CRP as a continuous predictor and identified a dichotomous cutoff for high versus low CRP based on the Youden index. Baseline demographics and clinical characteristics were summarized by CRP category using medians with interquartile range or percentages as appropriate. Baseline characteristics were compared using the Kruskal–Wallis test or Cochran–Mantel–Haenszel test.

The primary outcome was modified Rankin ≥4 at 8 weeks of antitubercular therapy. This score was chosen as it was considered a “poor outcome” in line with feedback from the Infectious Diseases Institute Community Advisory Board. A score of 4 denotes moderately severe disability where one is unable to attend to one’s own bodily needs or walk unassisted. A score of 6 denotes death. We estimated unadjusted odds ratios (ORs) for modified Rankin ≥4 at 8 weeks among participants with “high” versus “low” baseline CRP. Adjusted ORs were estimated using multivariable logistic regression models, and the baseline covariates were selected a priori based on known predictors of mortality in TBM (certainty of TBM diagnosis, Medical Research Council grade, and HIV status). Subgroup analyses included unmasking TBM IRIS and definite TBM. In the presence of missing data, only complete cases were used for multivariable analysis. *P* values <.05 were considered significant. No adjustments were made for multiple testing. Data analysis was conducted using SAS Version 9.4 (SAS Institute, Cary, NC)

## RESULTS

From March 2021 to November 2023, we enrolled 178 adults with TBM, 75% (135 of 178) had baseline CRP results and 8-week modified Rankin available. Of the 135 participants, 46% (61 of 135) were female with a median age of 36 years (interquartile range [IQR], 30–42) and 83% (112 of 135) were diagnosed with HIV, with a median CD4 count of 79 cells/μL (IQR, 38–177). Three quarters of the participants (76%, 103 of 135) had altered mental state (Glasgow coma scale <15) at baseline. Other baseline characteristics are summarized in Table 1. The median baseline blood CRP level was 37 mg/L (IQR, 12–102).

**Table 1.** Baseline Characteristics and Outcomes of 134 Ugandan Adults Who Presented With Suspected Tuberculous Meningitis Stratified by Baseline Serum C-Reactive Protein Measurement

Baseline Characteristic	CRP <40 mg/L (N = 67)	CRP ≥40 mg/L (N = 67)	*P* Value
Age, y	36 (30–40)	36 (28–43)	.79
Female	36 (54%)	24 (36%)	.04
Glasgow coma scale <15	49 (73%)	53(79%)	.42
TBM severity grade			.003
MRC grade I	14 (21%)	9 (13%)	
MRC grade II	47 (70%)	36 (54%)	
MRC grade III	6 (9%)	22 (33%)	
People with human immunodeficiency virus	57 (85%)	54 (81%)	.49
CD4 count, cells/μL	85 (44–214)	62 (17–132)	.02
Positive urine tuberculosis diagnostic	16 (23.5%)	27(40.3%)	.04
ART status			.23
On ART ≤3 m	7 (12%)	10 (19%)	
On ART >3 m	13 (23%)	15 (28%)	
Not on ART	36 (63%)	25 (46%)	
Unknown	1 (2%)	4 (7%)	
Uniform TBM case definition classification			.10
Definite	21 (31%)	29 (43%)	
Probable	35 (52%)	34 (51%)	
Possible	11 (16%)	4 (6%)	
CSF protein, mg/dL	120 (90–175)	110 (79–147)	.12
CSF white cells, cells/μL	80 (<5–210)	45 (<5–165)	.32
Acellular CSF (<5 cells/μL)	21 (32%)	25 (39%)	.46
Rifampicin dose			
35 mg/kg dosing	31(46%)	37(55%)	.26
8-wk outcome			
Modified Rankin ≥4	21(34%)	41(66%)	<.001

Data are n (%) of participants with available data or median (interquartile range). *P* values are via Cochran–Mantel–Haenszel test or Kruskal–Wallis test. CD4 and ART status among people with human immunodeficiency virus only. Urine tuberculosis diagnostics included Alere Determine TB Lipoarabinomannan Antigen or Xpert MTB/RIF Ultra.

Abbreviations: ART, antiretroviral therapy; CRP, C-reactive protein; CSF, cerebrospinal fluid; MRC, Medical Research Council; TBM, tuberculous meningitis.

When assessed as a univariate continuous predictor, the serum CRP had an area under the ROC curve of 0.70 (95% confidence interval [CI]: .61–.79), improving to 0.76 (95% CI: .68–.84) with adjustment for rifampicin dose, certainty of TBM, Medical Research Council grade, and HIV status. Cut points of 24.5 mg/L and 40 mg/L had the highest Youden indices at 1.31 and 1.31, respectively. A cut point at 40 mg/L had a better balance of specificity and sensitivity of predicting 8-week modified Rankin ≥4, with specificity at 64% and sensitivity at 66%. The corresponding values for the 24.5-mg/L threshold were 53% specificity and 77% sensitivity. A higher proportion of participants with CRP ≥40 mg/L (22 of 67, 33%) presented with severe TBM (MRC grade 3) versus 9% (6 of 68) with CRP <40 mg/L (Table 1).

At 8 weeks, 46% (62 of 135) of the study population had a modified Rankin ≥4. Supplementary Figure 1 illustrates the distribution of baseline CRP in the study population stratified by disability status (modified Rankin ≤3 versus ≥4). Among participants with modified Rankin ≥4 at 8 weeks, 61.2% (41 of 67) had CRP ≥40 mg/L at baseline versus 30.9% (21 of 68) of participants with modified Rankin ≤3 at 8 weeks. The odds of having modified Rankin ≥4 at 8 weeks were 3-fold higher among participants with baseline CRP ≥40 mg/L compared with those with baseline CRP <40 mg/L (unadjusted OR, 3.53; 95% CI: 1.73–7.19; *P* < .001). The association remained after adjusting for rifampicin dosing, certainty of TBM diagnosis (definite versus probable/possible), MRC severity grade, and HIV status (adjusted OR, 2.78; 95% CI: 1.28–6.04; *P* = .010).

The association of high baseline CRP with disability or death was not statistically different across prespecified subgroups based on suspected unmasking TBM IRIS and certainty of TBM diagnosis.

## DISCUSSION

Our data show that a heightened state of systemic inflammation in TBM, defined here as baseline serum CRP measurement of ≥40 mg/L, predicts severe neurological disability or death at 8 weeks. We are the first to explore CRP as a prognostic predictor in TBM. CRP could be useful in identifying individuals who may benefit from additional supportive care, for example, neurocritical care provision or additional host-directed therapies.

More than 80% of the study population had comorbid advanced HIV disease (median CD4 count <100 cells/μL). Given the meager (approximately 5%) overall survival benefit conferred by dexamethasone in HIV-associated TBM [7] and the known heterogeneity of immune response in this population, it may be valid to evaluate CRP-directed corticosteroids in HIV-associated TBM as the next step.

Findings from this study are consistent with those from a prior South African cohort of 81 patients with culture-confirmed HIV-associated pulmonary tuberculosis [14] in whom those with CRP >50 mg/L were more likely to have poor prognostic characteristics such as higher mycobacterial load and disseminated disease. Further, patients with disseminated tuberculosis who died had increased markers of innate immune activation compared with survivors [15]. Thus, elevated serum CRP may be a surrogate biomarker of a dysregulated detrimental immune response in disseminated tuberculosis. In our population, one third had positive TB-Lipoarabinomannan or urine Xpert MTB/RIF, with the higher CRP group contributing a significantly higher proportion (40% versus 24%).

This exploratory work had limitations. CRP elevation may also reflect bloodstream infections and bacterial sepsis, which are common in advanced HIV. We were not able to account for potential confounders such as bloodstream infections and other concomitant disease-causing systemic inflammation. Nevertheless, we believe that CRP elevation is highly likely due to active tuberculosis as the proportion of participants with CRP >40 mg/L was highest in those with definite (microbiologically confirmed) TBM followed by probable TBM.

In conclusion, our data indicate that a baseline CRP ≥40 mg/L may be a useful predictor of severe disability or death and could be a surrogate marker for immune-mediated damage in TBM. Serum CRP could be a useful biomarker to customize adjunctive host-directed therapy or target supportive care in TBM and should be further investigated.

## Supplementary Material

ciaf261_Supplementary_Data

## Supplementary Data

Supplementary materials are available at *Clinical Infectious Diseases* online. Consisting of data provided by the authors to benefit the reader, the posted materials are not copyedited and are the sole responsibility of the authors, so questions or comments should be addressed to the corresponding author.

## Notes

***Acknowledgments*.** We thank the HARVEST trial funders of the Department of Health and Social Care, the Foreign, Commonwealth & Development Office, the United Kingdom Medical Research Council, and the Wellcome Trust Medical Research Council Joint Global Health Trials (MR/S004963/1). This UK funded award is part of the European & Developing Countries Clinical Trials Partnership 2 program supported by the European Union. This work was also supported by the Fogarty International Center, the National Institute of Neurological Disorders and Stroke (R01NS086312, K23NS110470), and the National Institute of Allergy and Infectious Diseases at the National Institutes of Health (R01AI170158, K23AI138851, K24AI184270).

***Collaborators*.** Biyue Dai, Jane F. Ndyetukira, Cynthia Ahimbisibwe, Alisat Sadiq, Florence Kugonza, Shifa Nabbale, Abdu K. Musubire, Jane Gakuru, Derrick Kasozi, Isaac Turyasingura, Morris K. Rutakingirwa, Edward Mpoza, John Kasibante, Kenneth Ssebambulidde, Tadeo Kiiza, Alice Namudde, Andrew Akampuira, Tony Luggya, Richard Kwizera, Michael Okiror, Dora Babirye, Rhona Muyise, John Kisembo, Andrew Luswata, Stewart, and Ananta Bangdiwala.

***Financial support.*** L. T. was supported by the Fogarty International Center U.S. National Institutes of Health (NIH) under grant D43TW009345 awarded to the Northern Pacific Global Health Fellows Program. J. E. was supported by a Wellcome Trust Clinical PhD Fellowship (203905/Z/16/Z). F. V. C. was supported by a Wellcome Early Career Award (300088/Z/23/Z).
