# Supplementary figures and images for: Prognostic Value of C-Reactive Protein in Adults With Tuberculous Meningitis: A Prospective Cohort Study
Source: Clin Infect Dis. 2025 Jun 24;81(5):e410–3. doi: 10.1093/cid/ciaf261 (PMC12728270; doi:10.1093/cid/ciaf261)

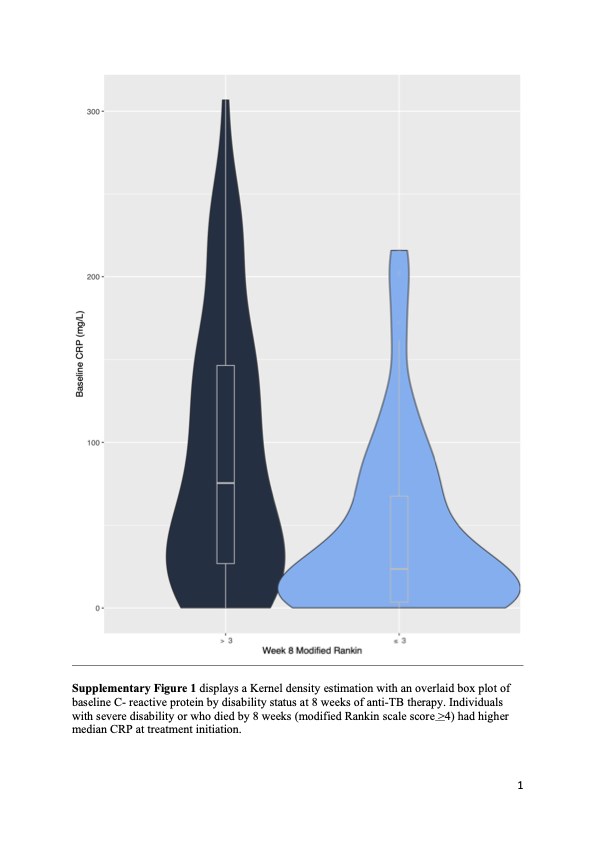

Supplement: ciaf261_Supplementary_Data [file ciaf261_supplementary_data.jpeg]
